# The Effects of Cadmium Exposure on the Oxidative State and Cell Death in the Gill of Freshwater Crab *Sinopotamon henanense*

**DOI:** 10.1371/journal.pone.0064020

**Published:** 2013-05-30

**Authors:** Jinxiang Wang, Pingping Zhang, Qingqing Shen, Qian Wang, Dongmei Liu, Jing Li, Lan Wang

**Affiliations:** 1 School of Life Science, Shanxi University, Taiyuan, People's Republic of China; 2 School of Life Science, Shanxi Datong University, Datong, People's Republic of China; Instituto de Tecnologica Química e Biológica, UNL, Portugal

## Abstract

We studied here the short-term toxicity effects of Cd on the oxidative state and cell death in the gill of freshwater crab *Sinopotamon henanense*. Crabs were exposed to Cd that resulted in Cd accumulation and a significant increase in the metallothionein (MT) level in the gill, but MT level increased disproportionally compared to the Cd accumulation with an extension of exposure time. Significant changes in the activities of superoxide dismutase (SOD), catalase (CAT), and glutathione peroxidase (GPx) were observed. An increase in the levels of reactive oxygen species (ROS) and lipid peroxidation (LPO) was detected that will cause oxidative stress. Histological abnormalities of the gills were discovered, including the expansion of gill cavity, a decrease in the numbers of connection of the upper and the lower of the gill lamellae and epithelial cells, and an increase in the number of hemocytes. The results of a TUNEL test and transmission electron microscope (TEM) showed that more gill cells had apoptotic characteristics after 48 h of Cd treatment compared to the control, but epithelial cell necrosis and inflammatory response appeared only after 72 h. It was concluded that (1) Cd induced the ROS production and accumulation through inhibiting antioxidant enzyme activities and exceeding the saturation values of MT binging; (2) Cd led to lipid peroxidation and histopathological alternations; and (3) Cd induced apoptotic response at short time exposure, followed by necrotic features and inflammatory reaction after longer time exposure.

## Introduction

Heavy metal pollution of the aquatic environment may be caused by natural and anthropogenic activities. Cadmium (Cd), one of the toxic heavy metals, enters aquatic organisms by their food and from the ambient environment through gills and epithelia where it is taken up through calcium channels of the plasma membrane of cells [1]. Cd not only induces DNA strand breaks, chromosome aberrations [2] and gene expression alterations [3], but leads also to tissue damages [4]–[6], morphological deformities [7], and even death [8]. The mechanisms of Cd damage are still insufficiently known.

One aspect of cadmium toxicities is the generation of cytotoxic reactive oxygen species (ROS) that cause oxidative deterioration of biological macromolecules [9]. In cells, there is a balance between ROS production and antioxidant defense which is an important removal mechanism of reactive oxygen species. Superoxide dismutase (SOD), catalase (CAT), and glutathione peroxidase (GPx) constitute a part of the antioxidant system that protects cells against ROS. O_2_^•—^ is scavenged by SOD and H_2_O_2_ is decomposed by GPx and CAT. When the rate of ROS generation exceeds the antioxidant capacity of cells, severe oxidative stress will result in oxidative damage. In addition to the enzyme index, a central measure of oxidative stress is lipid peroxidation (LPO), as indicated by malondialdehyde (MDA) levels, which can accumulate as a consequence of cellular damage [10], [11].

Metallothioneins (MTs), small cysteine-rich proteins, are the most abundant intracellular metal-binding proteins. MT is induced by and binds to Cd, and is then stored as a nontoxic Cd-MT complex in organism [12]. MT also acts as radical scavengers to protect cells from an array of stress responses [13], [14]. Cells with more MT are protected against heavy metal toxicity and oxidative stress, whereas under-expression in cell lines they lead to elevated sensitivity to Cd resulting in oxidative stress [15].

Cadmium-induced cellular toxicity has been related to necrosis and/or apoptosis [8], [16], [17]. Necrosis is quite different from apoptosis. Necrotic cells first swell, and then the plasma membrane collapses and cells are rapidly lysed. Apoptotic cells first shrink and their nuclei get condensed, then they disintegrate into well-enclosed apoptotic bodies [18]. Cell apoptosis is self-destruction without any inflammatory reaction. By contrast, necrosis might have important biological consequences, including the induction of an inflammatory response [19]. Cd has been reported to induce rainbow trout hepatocyte apoptosis [20], necrosis in the crustacean heart [6] and apoptosis or necrosis in U937 cells [21]. All these damages are related to oxidative stress and are proportional to the concentration of oxidants. Troyano et al. [22] suggested that the duration of the oxidative state seemed to be critical in determining the mode of death including apoptosis and necrosis.

The freshwater crab *Sinopotamon henanense* lives close to sediments and is reported to easily accumulate Cd which leads to oxidative damage and tissue structure abnormalities of heart and testis [6], [23], [24]. Cytotoxic studies also showed that Cd-induced apoptosis in gills is related to the production of ROS [25]. But the harmful effect of Cd on gill structure and the mode of Cd-induced cell death are as yet unclear in freshwater crab. In the present study, we investigated short-term toxicity effects of acute Cd exposure on the oxidative state, histological structure and cell death (apoptosis and necrosis) in the gill.

## Materials and Methods

### Chemicals and apparatus

All chemicals used in the present study were analytical grade and obtained from Sigma Co. (St. Louis, MO, USA). Assay kits for Hydrogen peroxide and TUNEL test were purchased from Beyotime Institute of Biotechnology (Haimen, Jiangsu Province, China).

### Animal material and treatments

Freshwater crabs, *S. henanense*, were obtained from the Dongan aquatic market in Taiyuan, China. Crabs were acclimated for 2 weeks in glass aquaria prior to the experiments and fed commercial feed three times a week. Only healthy adult male crabs with a homogeneous weight (20.0±0.5 g) were used. The crabs were divided into 3 groups that were exposed to CdCl_2_ solution. The nominal exposure concentration of Cd were 14.5, 29 and 58 mg·L^−1^ (corresponding to 1/16, 1/8, 1/4 of the 96 h-LC_50_) for 96 h [26]. Crabs were treated at the same conditions for the whole period and served as controls for the above. During the experiment, crabs were not fed and after treatments, the gills of crabs were sampled and stored at −80°C for further analysis. The treatment protocol is shown in Table 1.

**Table 1:** Treatment protocol of *Sinopotamon henanense* in different groups.

Groups	Control	Group A	Group B	Group C
Nominal exposure concentration of Cd (mg·L^−1^)	0	14.5	29	58

### Cd accumulation assay in gill and Cd concentration assay in water

At 12, 24 48, 72 and 96 h of acute Cd exposure, the gill tissues were excised, freshly weighed, and washed in a PBS buffer (pH 7.2). Later tissues were digested in HNO_3_ and HClO_3_ at about 120–150°C. The water samples from each group were collected and filtered after 0, 24, 48, 72 and 96 h of acute Cd exposure. Cd concentrations were measured with an atomic absorption spectrophotometer (SHIMADZU AA-6300, Japan). The Standard Cd Solution (Shanxi Environmental Protection Department) was used as an analytical control for metals. The carrier gas was argon and the internal standard was rhodium. Cd concentration was expressed as µg·g^−1^ wet weight tissue.

### MT Measurement

MT was determined using the methods as described by Li et al. [27] and Ma et al. [23]. Sampling tissues were freshly weighed, and then gently homogenized in extraction buffer (10 mM Tris, pH 8.0, 0.1 mM PMSF and 0.1 mM DTT) on ice. After centrifugation at 16,000 g for 30 min at 4°C, the supernatant was heated for 2 min in a boiling water bath and centrifuged at 10,000 g for 10 min to remove precipitated proteins. Volumes of 0.1 mL Cd solution (500 µg·L^−1^ as CdCl_2_) were mixed with 0.5 mL of the supernatant and incubated at room temperature for 10 min, and 0.5 mL of a 2% (w/v) bovine hemoglobin solution was then added and incubated at room temperature for 10 min. The hemoglobin was denatured at 100°C for 2 min, cooled in ice for 3 min, and centrifuged at 10,000 g for 15 min. The supernatants were transferred into clean tubes. Steps from the addition of the bovine hemoglobin solution until centrifugation were repeated three times. The amount of Cd ions in the final supernatant was proportional to the amount of MT present. The concentration of Cd in the supernatant was determined using an atomic absorption spectrophotometer (SHIMADZU AA-6300, Japan). The estimated concentration of MT was calculated by the following equation: MT (µg·g^−1^ w wt) = Cd (µg·g^−1^ w wt)/112.4/6×6000. According to Pedersen et al. [28] and Schlenk et al. [29], 1 mol crab MT was bound to 6 mol metal ions and the crab MT average molecular weight was assumed to be 6000 Da. MT concentration was expressed as µg·g^−1^ wet weight tissue.

### Determination of MDA contents and activities of SOD, GPx and CAT

Frozen samples were homogenized (10% w/v) in 0.1 M PBS at 4°C. The homogenate was centrifuged at 3,000 g for 15 min at 4°C, and the supernatant was used for the following assay. Lipid peroxidation was measured using the thiobarbituric acid test for MDA according to Ohkawa et al. [30], based on the reaction between MDA and thiobarbituric acid (TBA) at 90–100°C to form a complex that absorbs maximally at 532 nm. Total SOD activity was measured by the xanthine/xanthine oxidase method [31]. One unit of SOD activity was defined as the amount of enzyme that inhibited the rate of NBT reduction by 50%. GPx activity was quantified as described by Rotruck et al. [32], based on the reaction between remaining glutathione after the action of GPx and 5,5′-dithio bis-(2-nitro benzoic acid) to form a complex that absorbs maximally at 412 nm. CAT activity was assayed by measuring the rate of decomposition of H_2_O_2_ at 240 nm [33]. Protein content was determined according to the method of Bradford [34] with bovine serum albumin as a standard.

### Determination of H_2_O_2_ content

Frozen gill segments (0.1 g) were homogenized in a 1∶9 (w/v) 50 mM pH 6.0 phosphate buffer at 4°C. The content of H_2_O_2_ was analyzed with the Hydrogen Peroxide assay kit (Beyotime, S0038) according to the manufacturer protocols. In brief, test tubes containing 50 µl test solutions were placed at room temperature for 30 min and measured immediately with a spectrophotometer at a wavelength of 560 nm. Absorbance values were calibrated to a standard curve generated with known concentrations of H_2_O_2_.

### Histological observation

Three crabs were randomly selected from each group after 0, 24, 48, 72, and 96 h of Cd exposure. Gill tissues were carefully excised and fixed in 4% buffered formalin for 24 h, followed by dehydration with ethanol and toluene series and embedded in paraffin. Approximately 4 µm-thick serial sections were obtained and stained with hematoxylin and eosin (H&E) for observation with a light microscope (Olympus BX51).

### TUNEL test

Paraffin tissue sections were mounted on slides for terminal deoxynucleotidyl transferase-mediated dUTP nick end labeling (TUNEL) assay following the kit instruction. Briefly, the sections were deparaffinized, hydrated and immersed in freshly prepared 3% hydrogen peroxide. Each slide was permeated with a 10 µg·ml^−1^ proteinase K solution. The slides were incubated for 30 min at 37°C in a reaction buffer containing terminal deoxynucleotidyl transferase (TdT) and dUTP-biotin, followed by 30 min at 37°C with the solution containing horseradish peroxidase- -conjugated streptavidin (HRP-Streptavidin) and, finally, 30 min at 37°C with the 3,3′-diaminobenzidine (DAB) substrate solution. The nucleus was counterstained with hematoxylin. In the light microscope, the apoptotic cell nucleus appeared as brown-yellow. Yet the non-apoptotic nucleus appeared blue. The DNase I-treated tissue was used as a positive control. The reaction without TdT enzyme was used as a negative control.

### Ultrastructural analysis with transmission electron microscopy

After the exposure period, crabs were rinsed and three to five tissue pieces from the middle of the gill lamellae (approximately 1 mm) were cut and then fixed in glutaraldehyde. After fixation, tissues were rinsed twice in buffer immediately and post-fixed in 1% osmium tetroxide, and then dehydrated in a graded ethanol series and embedded in thin viscosity resin. Ultrathin sections were cut with an ultramicrotome (Leica UC-6), stained with uranyl acetate and lead citrate, and examined using a transmission electron microscope (JEM-1011) at an accelerating voltage of 80 kV.

### Statistical analysis

Data were expressed as means ± SD and computed statistically using one-way analysis of variance (ANOVA). The post hoc least significant difference (LSD) test was performed for an inter-group comparison. Probability values of p<0.05 were considered as statistically significant.

## Results

### Cd concentration assay in water

The Cd concentration in water from each treatment group was analyzed during the experiment. The exposure experiment lasted for 96 h and the Cd concentration of the exposed groups was determined every day. As shown in Table 2, at 0 h, no significant changes in the Cd concentration from each treatment groups were observed compared to the nominal exposure concentration. However, the Cd concentrations in water in all groups treated with Cd declined significantly (*p*<0.05) after 24 h of acute Cd exposure compared with the nominal exposure concentration.

**Table 2:** Cd concentration analysis in water of freshwater crab (*S. henanense*) exposed. to Cd.

Group	Nominal exposure concentration (mg·L^−1)^	Measured exposure concentration in water (mg·L^−1^)				
		0 h	24 h	48 h	72 h	96 h
Control	0	0	0	0	0	0
Group A	14.50	14.35±0.29	12.75±0.86*	12.02±0.93*	11.55±1.22*	11.05±0.59*
Group B	29.00	28.55±0.26	27.27±1.04*	26.64±0.86*	25.71±0.26*	25.52±0.43*
Group C	58.00	57.25±0.54	55.46±0.77*	54.63±0.29*	54.01±0.67*	53.13±0.53*

Data are expressed as mean ± SD. Significance is shown by

* *P*<0.05, on comparing with respective nominal exposure concentration.

### Cadmium accumulation and MT induction

**Figure 1:**
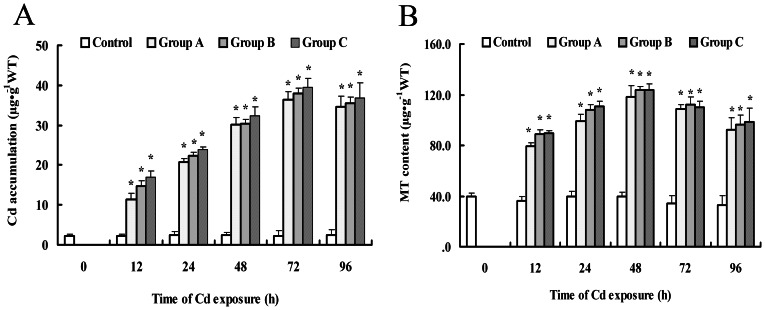
Accumulation of Cd and MT contents in the gills of *S. henanense* exposed to control, Group A, Group B and Group C. Data are means ± S.D. (n = 3). *Different from respective control (*P*<0.05).

Acute Cd exposure led to Cd accumulation in crab gills (Fig. 1A). Prior to these acute Cd exposure tests, the content of Cd in gills was about 2.25 µg Cd g^−1^ in the control group. Treatment with Cd led to a significant increase in Cd accumulation in any of the groups compared to the control within 12 h, maximized at 72 h, and then decreased at 96 h upon Cd treatment. The maximum value was 39.42 µg Cd g^−1^ in group C, which was 17.4-fold of the control. The data also demonstrated that the accumulation of Cd occurred in a concentration-dependent manner.

MT is a stress response protein. Our MT measurements showed that MT was induced rapidly by Cd at 12 h and the MT levels elevated to the highest values at 48 h and then declined at 72 h (Fig. 1B). The highest values of MT at 48h were 2.96, 3.1, 3.1-fold of the control for group A, group B and group C, respectively. And Cd-induced MT increased in a concentration-dependent manner after Cd treatment.

### Antioxidant defense responses

**Figure 2:**
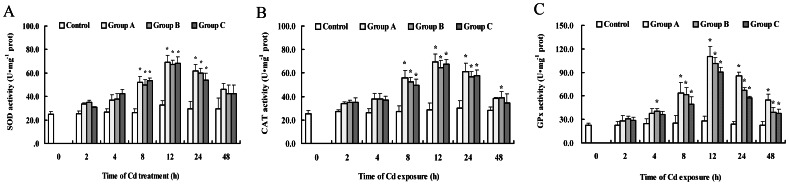
The effects of Cd on antioxidant enzyme activities of SOD (A), CAT (B) and GPx (C) in the gills of *S. henanense*. The values are means ± S.D. (n = 3). *Different from respective control (*P*<0.05).

As shown in Fig. 2, activities of antioxidant enzymes were determined over an experimental period of 48 h. During this period, enzyme activities of the control varied only slightly around their means (Fig. 2A, B, C). Treatment with high Cd concentrations resulted in the doubling of SOD activities at 12 h (Fig. 2A). After 12 h, SOD activities in gills decreased and remained higher than the control levels at 48 h. Enzyme activity increased initially significantly at 8 h for GPx, and reached the highest levels at 12 h (Fig. 2C). The activity of GPx increased maximally to 295% of the control in group A, followed by a decrease at 24 h in all Cd-treated groups. CAT activities also increased dramatically at 8 h of Cd exposure (Fig. 2B). However, this stimulation was only transient and the CAT activities dropped back to levels similar to those found in the control after 24 h in groups A and C.

### Oxidative stress indices

**Figure 3:**
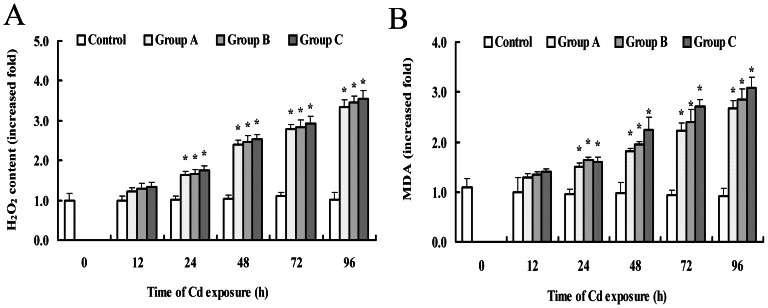
The effects of Cd on H_2_O_2_ content and lipid peroxidation in the gills of *S. henanense*. (A) H_2_O_2_ content; (B) MDA content. The mean expression in each treatment group is shown as a fold increase compared to the mean expression in the control, which had been ascribed an arbitrary value of 1. The values are the means ± S.D. (n = 3). Asterisks indicate a significant difference to the control (*P<0.05).

For the investigation of the hypothetical role of ROS formation due to Cd toxicity, levels of H_2_O_2_ were determined in crab gills at control conditions and in the presence of Cd (Fig. 3A). At control conditions, a small change of H_2_O_2_ was observed, most likely reflecting the basal rate of H_2_O_2_ formation that occurred as a by-product of aerobic metabolism. In the presence of Cd the production of H_2_O_2_ was significantly increased at 24 h in the treatment groups. The time-course analysis of H_2_O_2_ content showed that the lowest dose generated approximately 3.3 times more H_2_O_2_ than the control, while the highest dose generated approximately 3.5 times more H_2_O_2_ than the control at 96 h of exposure. It is evident that Cd exposure results in the robust generation of H_2_O_2_.

Lipid peroxidation levels in the gills of crabs, measured as the content of MDA, are given in Fig. 3B. At the absence of Cd, there were no significant changes in lipid peroxidation levels. However, with Cd there was a remarkable increase in lipid peroxidation levels which correlated positively with exposure time and concentration of Cd. The lipid peroxidation level increased to 147%, 163% and 185% of the control at 96 h for group A, group B and group C, respectively.

### Histological Analysis

**Figure 4:**
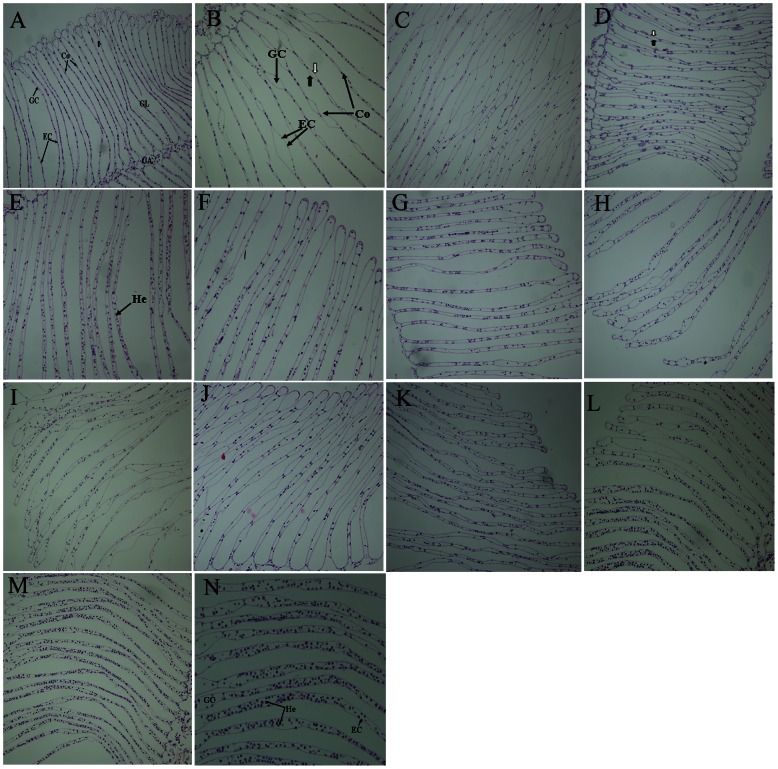
Histological analysis of Cd-induced gill injury in *S. henanense* by light microscopy. HE-stained gill section: A–M: 100×; N: 200×. (A) control; (B) exposure to Group A for 24 h; (C) exposure to Group A for 48 h; (D) exposure to Group A for 72 h; (E) exposure to Group A for 96 h; (F) exposure to Group B for 24 h; (G) exposure to Group B for 48 h; (H) exposure to Group B for 72 h; (I) exposure to Group B for 96 h; (J) exposure to Group C for 24 h; (K) exposure to Group C for 48 h; (L) exposure to Group C for 72 h; (M) exposure to Group C for 96 h; (N) exposure to Group C for 96 h. Co: connection of gill lamellae; EC: epithelium cells; GC: gill cavity; GL: gill lamellae; GA: gill axisx; He: hemocyte.

During the experiment no crab died and no remarkable pathological changes were observed in gills studied in the control after microscopic examination (Fig. 4A). Crabs exhibited a normal gill structure, including slender gill lamellae, regular and dense epithelium cells, the upper (bold white arrow) and lower monolayer cell (bold black arrow) of the gill lamellae connected through epidermal cells and a relatively small amount of hemocytes in the gill cavity. Microscopic examination of the HE-stained gill sections of crabs exposed to Cd for 24 h revealed a slight degenerative process in comparison to the control group (Fig. 4B, F, J). A part of the connections of the upper and lower monolayer cell appeared separated, and the gill cavity appeared enlarged. After 48 h of Cd exposure, the number of connections between the upper and lower monolayer cells of gill lamellae and epidermal cells decreased (Fig. 4C, G, K). After 72 h exposure hyperemic lamellae with edema and even inflammatory foci were detected in all treatment groups as manifested by the presence of a large quantity of hemocytes in the gill cavity (Fig. 4D, H, L). When the exposure time was extended to 96 h (Fig.4E, I, M, N), the number of epidermal cells decreased, the gill cavity further enlarged and a large amount of hemocytes appeared in the gill cavity, indicating that the longer the exposure time, the more serious the tissue damage was.

The effects of continuous Cd exposure on the histological structure of gills were found to be concentration-dependent. The group with highest exposure to Cd showed clearly abnormal histopathology, which corresponded to an irregular arrangement of epidermal cells, the overall presence of fewer and less densely arranged cells, and the infiltration of dense inflammatory cells (Fig. 4J–N). Histology of gill tissue from crabs in groups A and B represented a transition between the control and the highest Cd concentration group (Fig. 4B–I). The abnormal histopathology was less severe in group A with less extensive areas of cellular loss, smaller gill cavity edema and fewer hemocytes compared to the highest Cd concentration group (Fig. 4B–E).

### TUNEL assay

**Figure 5:**
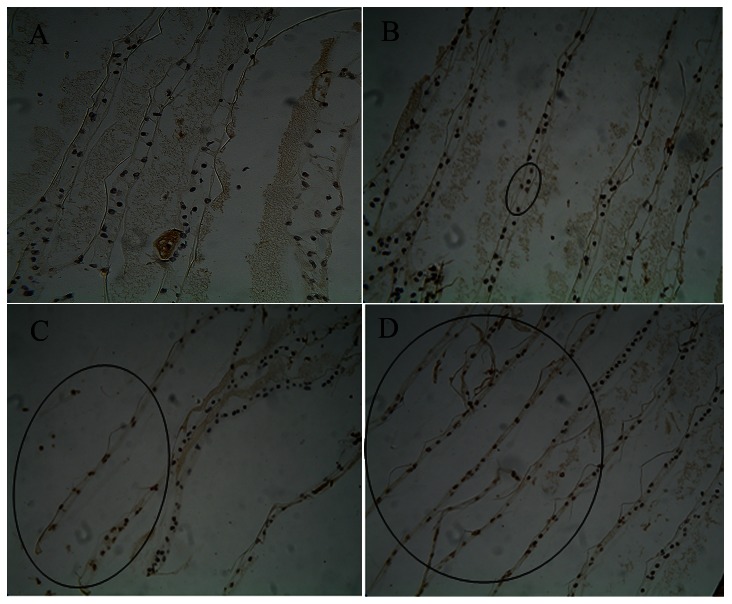
TUNEL test of Cd-induced apoptosis in gills of *S. *henanense. Stained gill section 200×. (A) control; (B) Group A; (C) Group B; (D) Group C. The apoptotic cellular nucleus was brown (circle), non-apoptotic cellular nucleus was blue.

Using TUNEL test, which labels fragmented DNA, two kinds of cells were recognized under light microscopy: apoptotic cells (brown-yellow by DAB staining in cell nucleus) and non-apoptotic cells (blue by hematoxylin counterstaining in cell nucleus). In the present study, TUNEL assays were performed to detect the mode of cell death in gills at 48 h of Cd exposure (Fig. 5). Microscopic examination of the TUNEL-stained sections showed that there were no positive cells in the control group (Fig. 5A). After 48 h of Cd treatment, positive cells were observed in the treatment groups. With increasing Cd concentrations, the number of apoptotic cells increased (Fig. 5B–D). Crabs exposed to highest concentrations of Cd (group C) contained a large number of TUNEL-positive, apoptotic cells in the gill epithelium (Fig. 5D).

### Ultrastructural analysis

**Figure 6:**
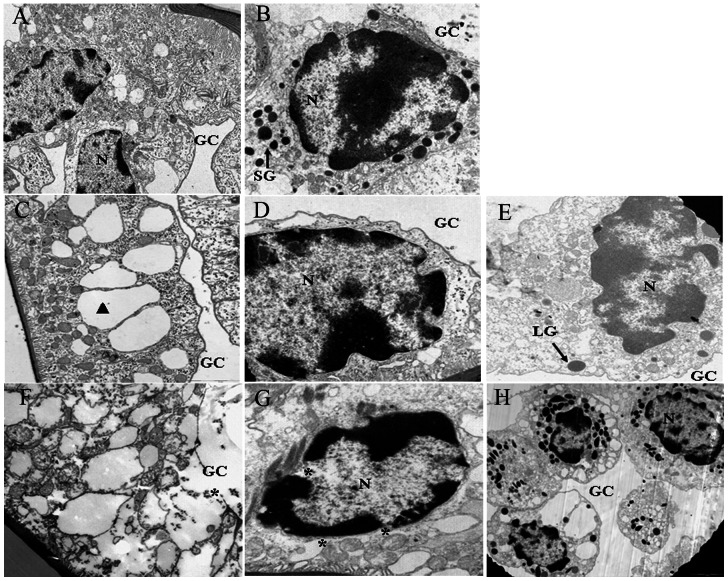
Effects of Cd on subcellular structure of *S. henanense* gills. A–B: control. (A) normal epithelial cell with nucleus and a large number of cytoplasmic organelles; (B) normal hemocyte in the gill cavity. C–E: exposure to Group C for 48 h. (C) cytoplasm of epithelial cell with the vacuolar enlargement (black triangle) and organelles reduction; (D) nucleus of epithelial cell with chromatin condensation and extremely irregular nuclear membrane; (E) hemocytes in the gill cavity with apoptotic characteristic. F–H: exposure to Group C for 96 h. (F) cytoplasm of degenerative and necrotic epithelial cells with scare organelles and broken membrane (asterisks); (G) nucleus of necrotic epithelial cells with nuclear membrane collapses (asterisks); (H) hemocytes in the gill cavity with inflammatory response. A: 6000×; B: 8000×; C: 8000×; D: 10000×; E: 10000×; F: 8000×; G: 8000×; H: 5000×. N: nucleus; LG: large granules; SG: small granules; GC: gill cavity.

The toxic effects of Cd on gill cells in group C were more evident in the ultrastructural study. As shown in Fig. 6, in the control group, epithelial cells showed a large number of organelles (Fig. 6A). Heterochromatin in the nucleus was uniformly distributed and adherent to the intact nuclear envelope (Fig. 6B). The apoptotic characteristic of both epithelial cells and hemocytes was observed at 48 h after Cd exposure (Fig. 6D, E). The nuclear member was intact with chromatin condensation and extremely irregular nuclei. Additionally, the vacuoles (▴) were enlarged and organelles were reduced in epithelial cells, and the hemocytes in the cavity were fewer (Fig. 6C, E). With the prolongation of exposure time to 96 h, the toxic effects of Cd on gill cells were more serious and some of them suffered necrosis as judged by the lack of epithelial cells, gill cavity expansion, disappearance of organelles, the disruption of plasma membrane, mitochondrial membrane, and nuclear membrane (asterisks), and an inflammatory response, which indicated necrotic cell death (Fig. 6F–H). This inflammatory phenomenon was judged by a large amount of hemocytes in the gill cavity (Fig. 6H).

## Discussion

Cadmium is taken up from the water in freshwater fish or crab mainly via the gills, and then it is transferred across the apical membrane and accumulated in gill cells [1]. Accumulated gill Cd could not be removed by ethylene diamine tetraacetic acid (EDTA) challenge [35], suggesting that gill cells have a limited ability for Cd excretion and may concentrate extremely high levels of Cd. Therefore, the bioaccumulation of Cd by *S. henanense* was first investigated. In this study, Cd accumulated in a time- and concentration-dependent manner and during the 96 h exposure period, the maximum values of accumulation in all treatment groups occurred within 72 h. These observations further supported an earlier study of Ma et al. [23], which demonstrated that the content of Cd continued to increase within 72 h. Low Cd concentration exposure (0.005–5 mg·L^−1^) experiment also confirmed that the amount of Cd accumulated in gill showed an increase with longer exposure [36]. After 24 h of acute Cd exposure, Reduction of Cd concentration in water from treatment groups indicated that Cd from the ambient water was transferred to the body of the crabs.

The degree of cell damage under heavy metal stress depends on the efficiency and capacity of detoxification and repair mechanisms [24]. The production of MTs has evolved as a mechanism to regulate metal levels and distribution within cells and organisms. The increase in protein content further proves that MT is involved the detoxification function of heavy metals. Our investigation showed that the content of MT increased with increasing concentration within the ambient medium and exposure time within 48 h. This suggests that MT is induced to reduce the level of toxic Cd ions in gill cells via binding to Cd, and to decrease the oxidative damage via scavenging ROS. Although Cd exposure clearly induced MT expression, its synthesis was not proportional to Cd accumulation at a later stage of cadmium exposure, e.g, the Cd accumulation increased but the MT level decreased after 48 h. The results were consistent with the findings of Ma et al. [23], which demonstrated that MT levels elevated rapidly to the highest values at 24 h and then declined at 72 h. The data presented here indicated that oxidative stress and cell damage were more serious after 48 h of exposure because the uptake of Cd exceeded the detoxification capacity of MT.

In addition to MT, is the antioxidant defense system keeping the routinely formed ROS at a low non-toxic level [37]. Cd treatment increased GPx and CAT activities before 24 h, respectively, indicating that antioxidant mechanisms are stimulated and can effectively scavenge ROS to maintain a normal cellular balance. The activities of CAT and GPx decreased after 24 h in all treatment groups, suggesting that excessive Cd accumulation resulted in a substantial inhibition of the antioxidant response and the accumulation of oxidative substances. Cd promoted an initial increase followed by a decrease of SOD. The changes of antioxidant enzyme activities explained changes in the H_2_O_2_ level, which had no difference compared with the control at 12 h of the treatment but increased significantly after this period. These results support the notion of the “adaptive stage” and the “inhibitive stage”, which proposes that the induction of antioxidant enzymes at the initial exposure time could efficiently attenuate the accumulation of H_2_O_2_ and maintain a normal cellular balance, whereas the later inhibitory state renders the enzyme unable to sufficiently scavenge the H_2_O_2_, leading to oxidative damage [10].

Cadmium is known to induce ROS, thereby causing lipid peroxidation [24], [38]. Long-term exposure to Cd disrupts the equilibrium between ROS generation and detoxification. Thus, ROS are generated following acute Cd intoxication and play important roles in tissue damage [39]. MDA levels are commonly accepted as an effective biomarker of toxic pollutant exposure [10]. In the present study, H_2_O_2_ levels increased significantly at 24 h which correlated with the decrease in antioxidant enzyme activities. A lack of increase in MDA content before 24 h may indicate negligible tissue damages caused by oxidative radicals, which might be due to the protective effects of the antioxidant system and MT. With the constitutive increase of an Cd-induced H_2_O_2_ content, a general trend for the induction of MDA can be observed. Low basal enzyme activity after 24 h treatment appeared to impair the ability of scavenging free radicals in animals and, in turn, resulted in elevated MDA levels. The MDA level increase was followed by consistent decreases in antioxidant enzyme activities. Based on these observations, we can infer that a variation in membrane damage may represent different toxicities exhibited by different exposure durations. The longer the exposure time, the more severe are cells subjected to oxidative stress and damage.

Oxidative stress provides a threat to cells. H_2_O_2_ activates the apoptotic response at the beginning of peroxide exposure, whereas inhibition of apoptosis leads to necrosis when the apoptotic process can be inhibited [21]. It was reported that Cd caused an intracellular stable accumulation of peroxide oxidation and death by apoptosis in U-937 cells, and pre-incubation with BSO, a GSH-depleting agent, switched the mode of death from apoptosis to necrosis in Cd-treated cells [16]. So a modulation of antioxidant defense systems in cells affects form and intensity of cell death [40]. Cell death resulting from Cd intoxication has been confirmed to induce both necrosis and apoptosis. The ratio between both types of cell death is dose- and cell-type-dependent [41]. Our previous study showed that Cd induced an apoptotic response in gill cells of freshwater crab depending on the H_2_O_2_ production [25]. But necrotic phenomena were not reported here. In the present study, HE stain, TUNEL and TEM figures further showed that Cd treatment led to damages of cell in gill, including the separation of the upper and lower monolayer cells of gill lamellae, edema in gill lamellae, enlargement of the gill cavity at 24 h, abnormity of gill lamellae and apoptotic cell death with nuclei condensation of epithelial cells at 48 h, necrotic cell death of epithelial cells with the decrease in the number of epithelial cells, gill cavity swelling, and the induction of an inflammatory response at 72 and 96 h. The above results suggest that the duration of Cd exposure and the oxidative state seem to be critical in determining the mode of death. The result was consistent with that of Ishido et al. [42], who demonstrated that Cd induced apoptotic chromatin condensation at an early stage of exposure, followed by necrotic features of the cells.

A transient oxidation may suffice to trigger apoptosis. By contrast, necrosis requires not only an increase in intensity, but also that the oxidative state stays on. Several reports confirm that excessive oxidation may deplete the cells in ATP, prevent the induction of apoptosis and drive the cells into necrosis [16], [40]. Besides, inactivating caspases provoked by excessive ROS and MT could play a role in controlling the choice between apoptosis and necrosis [41], [43], [44]. At any rate, Cd led to oxidative stress and cell death in crab gills. Prolonging exposure time of Cd failed to further increase apoptosis but resulted in elevated necrosis.

## Conclusion

Acute Cd exposure leads to the accumulation of Cd in the crab gill. At the initial state of Cd exposure, as one of the important detoxification mechanisms, MT and the antioxidant defense system are activated and decrease the free Cd ion level and caused a low cellular ROS content initially at 24 h. With increased exposure time, the toxicity of Cd exceeded the detoxification capacity of MT and that of the antioxidant defense system, which resulted in the accumulation of ROS. Once cell damage became more serious, lipid peroxidation and histopathological alternations were observed after 24 h. The cells appeared apoptotic after 48 h of Cd exposure, and exhibited necrotic and inflammatory characteristics after 72 h exposure time.
